# Konzeption und Implementierung eines Zertifizierungssystems zur Qualitätssicherung der Cochlea-Implantat-Versorgung in Deutschland

**DOI:** 10.1007/s00106-023-01305-x

**Published:** 2023-04-28

**Authors:** T. Stöver, S. K. Plontke, O. Guntinas-Lichius, H-J. Welkoborsky, T. Zahnert, K. W. Delank, T. Deitmer, D. Esser, A. Dietz, A. Wienke, A. Loth, S. Dazert

**Affiliations:** 1 Klinik für Hals‑, Nasen‑, Ohrenheilkunde, Universitätsklinikum Frankfurt, Theodor-Stern-Kai 7, 60590 Frankfurt, Deutschland; 2 Klinik für Hals‑, Nasen‑, Ohrenheilkunde, Kopf- und Halschirurgie, Universitätsmedizin Halle, Halle (Saale), Deutschland; 3 Klinik für Hals‑, Nasen- und Ohrenheilkunde, Universitätsklinikum Jena, Jena, Deutschland; 4 Klinik für Hals‑, Nasen- und Ohrenheilkunde, Klinikum Nordstadt, Hannover, Deutschland; 5 Klinik für Hals‑, Nasen- und Ohrenheilkunde, Universitätsklinikum Dresden, Dresden, Deutschland; 6 Hals-Nasen-Ohren-Klinik, Klinikum Ludwigshafen, Ludwigshafen, Deutschland; 7 Klinik für Hals‑, Nasen‑, Ohrenheilkunde, Universitätsklinikum Leipzig, Leipzig, Deutschland; 8 Kanzlei WBK, Rechtsanwalt Fachanwalt Medizinrecht, Köln, Deutschland; 9 Klinik für Hals‑, Nasen- und Ohrenheilkunde, Universitätsklinikum (St. Elisabeth Hospital), Bochum, Deutschland

**Keywords:** Rehabilitation, Implantierbare Neurostimulatoren, Leitlinie, Prothesen und Implantate, Qualitätssicherung, Rehabilitation, Implantable neurostimulators, Clinical practice guidelines, Prostheses and implants, Quality control

## Abstract

Ein standardisierter und strukturierter Versorgungsprozess ist unabdingbare Voraussetzung für eine optimale Hörrehabilitation mit einem Cochlea-Implantat (CI). Auf Initiative des Präsidiums der Deutschen Gesellschaft für Hals-Nasen-Ohren-Heilkunde, Kopf- und Hals-Chirurgie e. V. (DGHNO-KHC) sollte auf Basis der aktuellen AWMF-Leitlinie (Arbeitsgemeinschaft Medizinisch-Wissenschaftlicher Fachgesellschaften), die den einheitlich in Deutschland geltenden medizinischen Standard in der CI-Versorgung beschreibt, ein Weißbuch erstellt und ein Zertifizierungssystem eingeführt werden, um die Umsetzung dieser Leitlinie standardisiert einrichtungsbezogen zu überprüfen und öffentlich zugänglich zu machen. Unter Einbindung einer unabhängigen Zertifizierungsorganisation sollte den beantragenden Kliniken durch die Erteilung des Qualitätszertifikats „Cochlea-Implantat-versorgende Einrichtung“ (CIVE) die erfolgreiche Umsetzung der Leitlinien- und Weißbuchinhalte bescheinigt werden können. Auf Grundlage der CI-Leitlinie wurde eine Struktur zur Umsetzung eines Zertifizierungsprogramms erarbeitet. Folgende Schritte waren hierzu notwendig: 1. Konzeption eines Qualitätssicherungssystems zur Zertifizierung leitlinienkonform arbeitender Kliniken; 2. Entwicklung der notwendigen Strukturen zur unabhängigen Überprüfung qualitätsrelevanter Struktur‑, Prozess- und Ergebnisparameter; 3. Erarbeitung eines Standardablaufs zur unabhängigen Zertifizierung von Kliniken; 4. Entwicklung eines Zertifikats und eines Logos zum Nachweis einer erfolgreichen Zertifizierung, 5. praktische Implementierung des Zertifizierungssystems. Nach Konzeption des Zertifizierungsprogramms, dessen Systematik und der dazugehörigen Organisationsstruktur wurde das Zertifizierungssystem 2021 erfolgreich eröffnet. Anträge zur Erteilung des Qualitätszertifikats CIVE konnten ab September 2021 formal gestellt werden. Bis Dezember 2022 wurden insgesamt 51 Offsite-Prüfungen durchgeführt. In den ersten 16 Monaten seit der Einführung wurden 47 Kliniken erfolgreich als CIVE zertifiziert. Im genannten Zeitraum konnten 20 Fachexperten als Gutachter geschult werden, die bis Dezember 2022 bereits 18 Vor-Ort-Prüfungen (Onsite-Audits) in Kliniken durchführten. Zusammenfassend konnte die Konzeptionierung, Strukturierung und praktische Umsetzung eines Zertifizierungssystems zur Qualitätssicherung in der CI-Versorgung für Deutschland erfolgreich umgesetzt werden.

**Zusatzmaterial online:**

Die Online-Version dieses Beitrags (doi:10.1007/s00106-023-01305-x) enthält ergänzend zur Abbildung 4 ergänzendes Zusatzmaterial in Form des vollständigen Erhebungs- und Kennzahlenbogens.

## Abstract (en)

A standardized and structured process is indispensable for optimal hearing rehabilitation with cochlear implants (CI). The Executive Committee of the German Society of Otorhinolaryngology, Head and Neck Surgery (DGHNO-KHC), initiated the conception of a certification system and a Whitepaper based on the Association of the Scientific Medical Societies in Germany (AWMF) clinical practice guideline (CPG) describing the current medical standards of CI care in Germany. The goal was to independently confirm implementation of this CPG and make this information publicly available. With the support of an independent certification organization, successful implementation of the CI-CPG by a hospital would then be verified and confirmed by awarding the “Cochlear implant-provision institution” (*Cochlea-Implantat-versorgende Einrichtung*, CIVE) quality certificate. A structure for implementation of a certification system was developed based on the CI-CPG. The following steps were required: 1) conception of a quality control system for certification of hospitals working in accordance with the CI-CPG; 2) development of required structures for an independent review of quality-relevant structure, process, and result parameters; 3) development of a standard procedure for independent certification of hospitals; 4) development of a certificate and a logo to demonstrate successful certification; 5) practical implementation of the certification. Following design of the certification system and the required organizational structure, the certification system was successfully launched in 2021. Applications for the quality certificate could be formally submitted from September 2021. A total of 51 off-site evaluations were performed by December 2022. In the first 16 months from introduction, 47 hospitals were successfully certified as CIVE. In this period, 20 experts were trained as auditors, who have since then carried out 18 on-site audits in hospitals. In summary, the conceptual design, structure, and practical implementation of a certification system for quality control in CI care was successfully implemented in Germany.

## Bedeutung der CI-Versorgung

Die Versorgung von hochgradig schwerhörigen oder ertaubten Menschen mit einem Cochlea-Implantat (CI) stellt seit vielen Jahren den „Goldstandard“ in der Hörrehabilitation betroffener Patienten dar [1]. Die CI-Versorgung ist ein sehr erfolgreicher, aber auch komplexer, zeitaufwendiger, kostenintensiver und lebenslanger Versorgungsprozess, der unter hals-nasen-ohrenärztlicher Leitung steht und nicht allein auf die chirurgische Implantation des CI begrenzt ist. Vor diesem Hintergrund ist sowohl ein interdisziplinärer und fachübergreifender Ansatz als auch die Sicherstellung der einzelnen diagnostischen, therapeutischen, rehabilitativen und qualitätssichernden Aspekte unabdingbar, um Patienten effektiv und sicher versorgen zu können.

Obwohl die Erfolge der CI-Versorgung unbestritten sind und seit mehr als 35 Jahren durch eine Vielzahl wissenschaftlicher Publikationen zweifelsfrei belegt sind, existiert eine erhebliche Schwankungsbreite im Hinblick auf die im Einzelfall erzielten Ergebnisse. Die Ergebnisse werden wesentlich von den individuellen Faktoren eines Patienten, wie z. B. der Ertaubungsdauer oder der Resthörigkeit, beeinflusst. Darüber hinaus werden die Resultate auch durch die chirurgische Technik, die Anpassung des Audioprozessors, die Rehabilitation und die Nachsorge bestimmt [2]. Versorgte Patienten benötigen eine interdisziplinäre Betreuung, die sich nicht allein auf rein medizinische Aspekte, wie die Chirurgie, bezieht, sondern u. a. audiologische, hör- und sprachtherapeutische, pädaudiologische, pädagogische sowie sozialmedizinische Aspekte betrifft.

Die optimale Nutzung des CI erfordert daher eine interdisziplinäre und lebenslange Betreuung implantatversorgter Patienten. Dies gilt sowohl für Kinder als auch für Erwachsene. Ein standardisierter und strukturierter Versorgungsprozess stellt die Voraussetzung eines bestmöglichen und lebenslangen Behandlungserfolgs dar. Ein weiteres Ziel dieser Strukturierung ist aber auch eine Risikominimierung in Bezug auf eine ungenügende Ergebnisqualität, wie z. B. eine unzureichende Hör- und Sprachentwicklung, eine fehlende oder ausbleibende Wiedererlangung der Teilhabe am alltäglichen Leben, aber auch medizinische Komplikationen. Auch führt eine ungenügend behandelte Einschränkung des Hör- und Sprachvermögens häufig zu einer erheblichen Beeinträchtigung der Lebensqualität [3].

## Medizinische Standards

Medizinische Standards werden i. d. R. mithilfe von medizinischen Leitlinien definiert und auf nationaler Ebene festgelegt. In Deutschland erfolgt dieses in einem standardisierten Verfahren über die Arbeitsgemeinschaft der Medizinisch-Wissenschaftlichen Fachgesellschaften (AWMF). Im Oktober 2020 wurde die Neufassung der Leitlinie CI-Versorgung unter Federführung der Deutschen Gesellschaft für Hals-Nasen-Ohren-Heilkunde, Kopf- und Hals-Chirurgie e. V. (DGHNO-KHC) erstellt. Diese Leitlinie stellt seitdem den in Deutschland geltenden Standard der CI-Behandlung dar (AWMF-Register-Nr. 017-071) [4].

Die aktuelle CI-Leitlinie umfasst erstmals wesentliche Aspekte der Qualitätssicherung im Hinblick auf Struktur‑, Prozess- und Ergebnisqualität in der CI-Versorgung. Als Beispiel kann hier die räumliche und technische Ausstattung, die fachliche Qualifikation, die Mindestanzahl der Mitarbeiter, die Sicherstellung des Anpassungs- und Rehabilitationsvorgangs sowie die Gewährleistung der lebenslangen Nachsorge für an einer Einrichtung versorgte Patienten erwähnt werden. Diese Leitlinie stellt damit einen Meilenstein in der Qualitätssicherung der CI-Versorgung in Deutschland dar.

Die Inhalte einer Leitlinie entsprechen dem aktuell geltenden Stand der Wissenschaft. Die Überprüfung der Einhaltung der Leitlinieninhalte war bislang, insbesondere für betroffene Patienten, nur unzureichend möglich. So existieren auf der einen Seite in Deutschland klare Empfehlungen für die Struktur‑, Prozess- und Ergebnisqualität der CI-Versorgung, und auf der anderen Seite fehlte bislang ein objektiver Nachweis der Umsetzung der Leitlinieninhalte. Diese Lücke sollte durch die Einführung des hier dargestellten Zertifizierungssystems geschlossen werden. Unter Einbeziehung einer unabhängigen, im Gesundheitswesen erfahrenen und entsprechend akkreditierten Zertifizierungsorganisation sollte den Kliniken durch die Erteilung des Qualitätszertifikats „Cochlea-Implantat-versorgende Einrichtung“ (CIVE) die erfolgreiche Umsetzung der Leitlinieninhalte bescheinigt werden können.

Auf Initiative des Präsidiums der DGHNO-KHC sollte daher ein deutschlandweites Qualitätssicherungssystem eingeführt werden, das erkennbar macht, welche Einrichtungen die aktuell gültigen Empfehlungen der Leitlinie umsetzen. Hierzu sollten folgende Ziele erreicht werden:

1. Konzeption eines Qualitätssicherungssystems zur Zertifizierung leitlinienkonform arbeitender Kliniken
2. Entwicklung der notwendigen Strukturen zur unabhängigen Überprüfung qualitätsrelevanter Struktur‑, Prozess- und Ergebnisparameter
3. Erarbeitung eines Standardablaufs zur unabhängigen Zertifizierung von Kliniken
4. Entwicklung eines Zertifikats und eines Logos zum Nachweis einer erfolgreichen Zertifizierung
5. Praktische Implementierung des Zertifizierungssystems

## Material und Methoden

### Erarbeitung der medizinisch-wissenschaftlichen Grundlagen der CI-Zertifizierung

Die Erstellung einer medizinisch-wissenschaftlich basierten Leitlinie erfolgt in Deutschland über einen standardisierten Prozess der Arbeitsgemeinschaft der Wissenschaftlichen Medizinischen Fachgesellschaften e. V. (AWMF). Aus diesem Prozess geht eine konsentierte Leitlinie hervor. Diese Leitlinie ist dann zu einem definierten Thema als die deutschlandweit geltende einheitliche Handlungsempfehlung für die Diagnostik, Therapie und Nachsorge für alle in diesem Feld tätigen Disziplinen zu betrachten.

#### CI-Leitlinie

Für die Erstellung der Leitlinie zur CI-Versorgung wurde die DGHNO-KHC als „federführende“ Gesellschaft von der AWMF beauftragt. Nachdem im Jahr 2001 die 1. Version der CI-Leitlinie verfasst wurde, konnte nach einem streng standardisierten Entwicklungsprozess im Oktober 2020 die aktuell 3. Fassung der CI-Leitlinie konsentiert und veröffentlicht werden [4]. Die aktuelle CI-Leitlinie wurde in enger Kooperation mit allen anderen medizinisch-wissenschaftlichen Fachgesellschaften, insbesondere der Deutschen Gesellschaft für Phoniatrie und Pädaudiologie (DGPP) und der Deutschen Gesellschaft für Audiologie (DGA), konsentiert.

#### CI-Weißbuch

Auf Basis dieser Leitlinie erfolgte wiederum unter Federführung der DGHNO-KHC die Erarbeitung des Weißbuchs „Cochlea-Implantat(CI)-Versorgung in Deutschland“ (CI-Weißbuch). Das CI-Weißbuch stellt eine praktische Handlungsanweisung für die Übertragung der Leitlinieninhalte in die klinische Praxis dar. Das CI-Weißbuch wurde ebenfalls mit der DGPP und der DGA fachlich konsentiert und im Mai 2021 durch das Präsidium der DGHNO-KHC verabschiedet und veröffentlicht [5].

### Schlüsselinhalte der CI-Leitlinie und des CI-Weißbuchs

**Figure 1:**
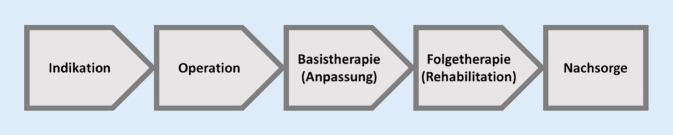

Das inhaltliche Ziel der CI-Leitlinie und damit des CI-Weißbuchs ist die Beschreibung relevanter Qualitätskriterien einer wissenschaftlich basierten CI-Versorgung. Dies schließt nicht nur die Implantation, sondern den gesamten Versorgungsprozess von der Indikationsstellung, der chirurgischen Versorgung, der Anpassung (Basistherapie), der Rehabilitation (Folgetherapie) und der Nachsorge ein (Abb. 1). Hierzu sind die Erfüllung der in der Leitlinie und im Weißbuch beschriebenen struktur-, prozess- und ergebnisrelevanten Qualitätsparameter die unabdingbare Voraussetzung einer erfolgreichen Hörrehabilitation CI-versorgter Patienten. Diese Parameter stellen damit in der Folge die Grundlage der erfolgreichen Zertifizierung einer Einrichtung dar.

Bei der Versorgung eines Patienten mit einem CI handelt es sich um einen interdisziplinären Prozess im engen Zusammenspiel unterschiedlicher Fachdisziplinen, z. B. HNO-Ärzte, Phoniater/Pädaudiologen, Audiologen, Neuroradiologen, Anästhesisten, Pädiater, Pädagogen, Logopäden, Sprachheiltherapeuten u. a. Die Endverantwortung für diesen Prozess liegt unzweifelhaft aufgrund medizinischer Aspekte (Indikationsstellung und Implantation) und auch rechtlicher Vorgaben in letzter Konsequenz in der Hand des HNO-Chirurgen bzw. an der Einrichtung, in der ein Patient mit einem CI versorgt wird (d. h. „Betreiber des Implantats“ laut „Medizinprodukte-Betreiberverordnung“ – MPBetreibV) [6]. Bei diesen Einrichtungen handelt es sich in Deutschland regelhaft um Kliniken (Hauptabteilungen) für Hals-Nasen-Ohren-Heilkunde, da nur diese alle notwendigen Strukturmerkmale aufweisen, um den in der Leitlinie und dem Weißbuch dargestellten Versorgungsprozess vollständig umsetzen zu können. Antragsberechtigt zur Erteilung eines CI-Zertifikats sind daher derzeit nur entsprechend ausgestattete und strukturierte Kliniken für Hals-Nasen-Ohren-Heilkunde. Um eine Differenzierung entsprechender Klinken zu ermöglichen, wurde in Deutschland der Begriff „Cochlea-Implantat-versorgende Einrichtung“ (CIVE) eingeführt. Dies war notwendig, da bislang keine einheitliche Verwendung anderer Bezeichnungen (z. B. CI-Zentrum, CI-Klinik usw.) erfolgte, die an die Erfüllung von Qualitätsanforderungen geknüpft war. Mit der Verwendung des Begriffs „CIVE“ erbringt die zertifizierte Einrichtung den Nachweis zur Erfüllung der nach Leitlinie und Weißbuch geforderten Qualitätsparameter. Dies bedeutet, dass eine Klinik, die die Bezeichnung „CIVE“ verwendet, erfolgreich gemäß den fachlich-wissenschaftlich definierten Anforderungen zertifiziert sein muss.

### Erarbeitung der inhaltlichen Grundlagen und Zielsetzung der Zertifizierung

Auf Basis der CI-Leitlinie und des CI-Weißbuchs sollte ein Fragenkatalog entwickelt werden, der die wesentlichen Anforderungen aus beiden Dokumenten erhebt. Dies war erforderlich, da die Leitlinie insgesamt 78 Seiten Text umfasst. Aus diesem Dokument sollte schließlich ein 35 Fragen/Items umfassender Katalog extrahiert werden. Dieser Erhebungs- und Kennzahlenbogen stellt damit die wesentlichen Parameter der Struktur‑, Prozess- und Ergebnisqualität des CI-Versorgungsprozesses zusammen. Die Angaben beruhen auf einer Ja- oder Nein-Antwort und der Bestätigung struktureller Vorgaben, wie z. B. der Qualifikation und der Anzahl von Mitarbeitern. Ein weiteres wesentliches Element ist die Sicherstellung der einzelnen Prozessschritte der CI-Versorgung, wie die Anpassung des Audioprozessors, die Sicherstellung der Rehabilitation sowie die lebenslange Nachsorge. Der Erhebungs- und Kennzahlenbogen ist damit die Grundlage des Zertifizierungsprozesses, der durch eine unabhängige Zertifizierungsorganisation überprüft wird.

### Formale Grundlagen zur Einrichtung der Zertifizierung

Das Präsidium der DGHNO-KHC hat nach Beschlussfassung im Jahr 2017 eine Arbeitsgruppe (Task-Force) eingerichtet, um das Thema Qualitätssicherung in der CI-Versorgung in Deutschland perspektivisch weiterzuentwickeln. Die dargestellten Arbeiten hatten damit einen mehrjährigen Vorlauf und stellen das Produkt intensiver konzeptioneller Arbeiten dar. Die fachliche Führung im Hinblick auf die medizinisch-wissenschaftlichen Inhalte der Zertifizierung lag beim Präsidium der DGHNO-KHC.

### Strukturelle Grundlagen der Zertifizierung

Um eine unabhängige Überprüfung der Qualitätskriterien gemäß der geltenden Leitlinie sicherzustellen, wurde durch die DGHNO-KHC eine unabhängige, im Gesundheitswesen erfahrene und akkreditierte Zertifizierungsorganisation (Fa. ClarCert GmbH, Neu-Ulm, Deutschland) beauftragt, die technische Umsetzung des Zertifizierungsvorgangs zu gewährleisten.

### Ablauf der Zertifizierung

**Figure 2:**
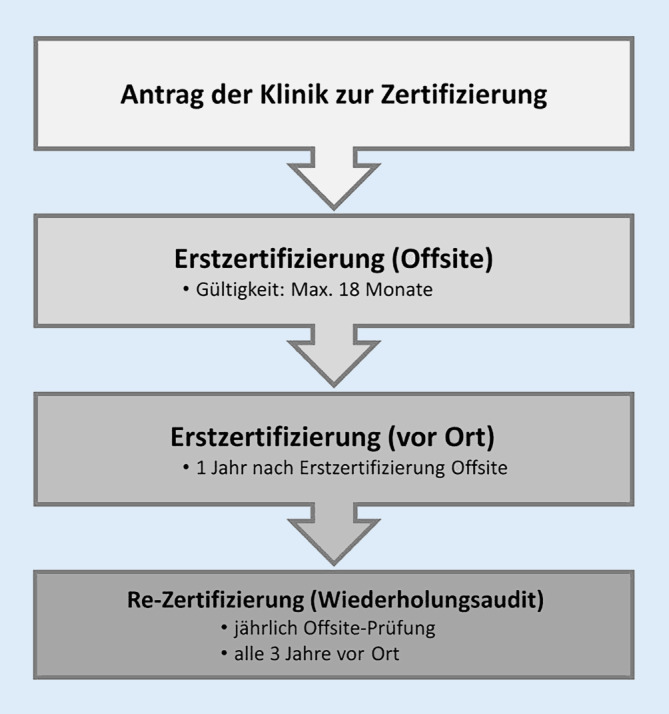

Der Zertifizierungsprozess unterliegt einem standardisierten Ablauf (Abb. 2). Das Verfahren beginnt mit der Antragstellung der zu zertifizierenden Einrichtung bei der Zertifizierungsorganisation. Diese Antragstellung umfasst die Bereitstellung der für die Zertifizierung kritischen Informationen zur Beurteilung der qualitätsrelevanten Struktur‑, Prozess- und Ergebnisaspekte. Diese werden anhand des Erhebungs- und Kennzahlenbogens schriftlich von der zu zertifizierenden Einrichtung zur Verfügung gestellt.

Die Erstzertifizierung erfolgt anhand der Bewertung des schriftlich eingereichten Erhebungs- und Kennzahlenbogens („Offsite-Prüfung“). Bei Erfüllung der notwendigen Voraussetzungen wird das Zertifikat zunächst für die Dauer von 18 Monaten erteilt. Das Zertifikat kann sowohl für die CI-Versorgung von Kindern *oder* Erwachsenen als auch für Kinder *und* Erwachsene beantragt und bei entsprechender Erfüllung der Anforderungen vergeben werden.

Die Bereitstellung des Erhebungs- und Kennzahlenbogens erfolgt nach der Erstzertifizierung einmal jährlich durch die CIVE, um mögliche strukturelle Veränderungen (z. B. Personalveränderungen) zu erkennen und für die Rezertifizierung zu berücksichtigen.

Beginnend mit dem ersten Jahr nach der Erstzertifizierung erfolgt eine Vor-Ort-Auditierung („Onsite“) durch Fachexperten. Die Aufgabe der Fachexperten besteht darin, die durch die CIVE im Erhebungs- und Kennzahlenbogen gemachten Angaben vor Ort zu verifizieren. Zudem sollen konstruktiv Verbesserungspotenziale aufgezeigt und im Bedarfsfall Abweichungen von den in der Leitlinie festgelegten Qualitätsstandards dokumentiert werden. Aus dem Bericht des Fachexperten ergibt sich eine Empfehlung zur Zertifikatserteilung. Ein Vor-Ort-Audit durch den Fachexperten erfolgt in Abständen von jeweils 3 Jahren zur Aufrechterhaltung des Zertifikats.

### Organisatorische Strukturen des Zertifizierungsprozesses

**Figure 3:**
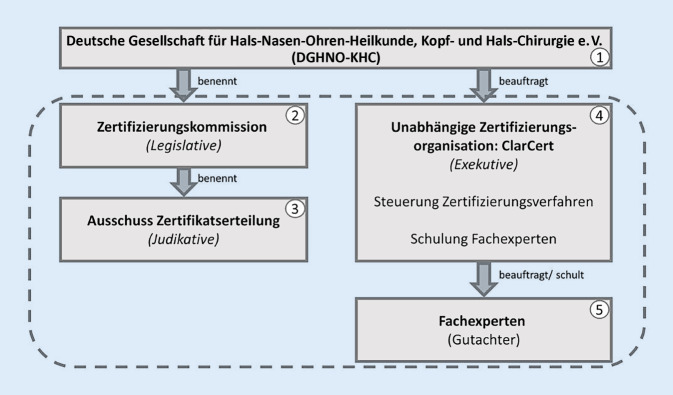

Um ein transparentes Vorgehen im Rahmen des Zertifizierungsprozesses sicherzustellen, bestand die Notwendigkeit zur Einrichtung organisatorischer Strukturen mit klar zugewiesenen Verantwortlichkeiten (Abb. 3). Im Einzelnen waren dies:

1. Die Deutsche Gesellschaft für Hals-Nasen-Ohren-Heilkunde, Kopf- und Hals-Chirurgie e. V. (DGHNO-KHC)
2. Die Zertifizierungskommission
3. Der Ausschuss Zertifikatserteilung
4. Die unabhängige externe Zertifizierungsorganisation
5. Die Fachexperten

#### Deutsche Gesellschaft für Hals-Nasen-Ohren-Heilkunde, Kopf- und Hals-Chirurgie e. V.

Die nationale medizinisch-wissenschaftliche Fachgesellschaft DGHNO-KHC erarbeitete im Konsens mit anderen im Bereich der CI-Versorgung tätigen Fachgesellschaften (besonders der DGPP und der DGA) die medizinisch-wissenschaftlich basierten Grundlagen für die aktuell gültige Leitlinie zur CI-Versorgung in Deutschland. Auf der Basis der Leitlinie wurde eine praktische Empfehlung zur Umsetzung der Leitlinie entwickelt, (CI-Weißbuch). Die fachliche Verantwortlichkeit für die Inhalte des Versorgungsprozesses lag damit abschließend bei der DGHNO-KHC.

#### Zertifizierungskommission

Die DGHNO-KHC benennt die Mitglieder einer Zertifizierungskommission, der die Funktion der „Legislative“ im Zertifizierungsprogramm zukommt. Die Aufgabe der Kommission besteht in der inhaltlichen Erarbeitung des Erhebungs- und Kennzahlenbogens auf Basis der CI-Leitlinie bzw. des CI-Weißbuchs. Der Erhebungs- und Kennzahlenbogen beinhaltet die wesentlichen Qualitätsaspekte der Leitlinie und stellt damit die Grundlage der Überprüfung der Struktur‑, Prozess- und Ergebnisqualität einer CIVE dar.

#### Ausschuss Zertifikatserteilung

Der Ausschuss Zertifikatserteilung nimmt die Aufgabe der „Judikative“ im Zertifizierungsprogramm wahr. Hierunter wird die fachliche Beurteilung der gestellten Zertifizierungsanträge verstanden. Diese Arbeitsebene setzt sich aus von der Zertifizierungskommission benannten Experten zusammen, die über die Annahme oder Ablehnung der Zertifizierungsanträge wie auch über die Zertifizierungsfähigkeit der antragstellenden Kliniken entscheiden.

#### Unabhängige externe Zertifizierungsorganisation

Die Aufgabenstellung der unabhängigen Zertifizierungsorganisation entspricht der „Exekutive“. Dies umfasst die technische Vorbereitung von Entscheidungen über Zertifizierungsanträge, die Überprüfung der formalen Antragsberechtigung, die Überprüfung der Erfüllung der formalen Struktur‑, Prozess- und Ergebnisvoraussetzungen (Erhebungs- und Kennzahlenbogen) sowie die Qualifizierung der Fachexperten zur Durchführung der Vor-Ort-Audits. Die Organisation überwacht ebenfalls unabhängig die formalen Schritte der Zertifizierung und die inhaltliche Erfüllung der von der Zertifizierungskommission vorgegebenen Qualitätskriterien an die Abläufe des Zertifizierungsprogramms.

#### Fachexperten

Die Aufgabe der „Fachexperten“ ist die Durchführung der Onsite-Audits vor Ort, zur Verifizierung der zuvor durch die antragstellende Einrichtung schriftlich gemachten Angaben sowie zur Benennung von Verbesserungspotenzialen oder Abweichungen in den Einrichtungen. Für diese Tätigkeit konnten sich Fachärzte für Hals-Nasen-Ohren-Heilkunde mit Erfahrung auf dem Gebiet der CI-Versorgung bei der Zertifizierungsorganisation bewerben. Die entsprechenden Qualifizierungskurse wurden durch die unabhängige Zertifizierungsorganisation durchgeführt, um die Fachexperten für ihre Tätigkeit als Gutachter fachlich und formal zu qualifizieren. Die erfolgreich qualifizierten Fachexperten werden dann im Verlauf des Zertifizierungsverfahrens von der Zertifizierungsorganisation beauftragt, um das Vor-Ort-Audit an der antragstellenden Einrichtung durchzuführen. Hierbei wird von den Fachexperten ein Audit-Bericht erstellt und eine Empfehlung zur Erteilung oder Nichterteilung des Zertifikats abgegeben. Außerdem kann eine Ablehnung oder auch eine Annahme unter festgelegten Auflagen durch den Fachexperten empfohlen werden. Die Empfehlung der Fachexperten stellt die Entscheidungsgrundlage für den Ausschuss Zertifikatserteilung dar, der abschließend über die Erteilung eines Zertifikats befindet.

### Zertifikat und Logo

Nach erfolgreicher Erstzertifizierung oder erfolgreicher Rezertifizierung durch ein Wiederholaudit vor Ort wird der antragstellenden Einrichtung über das Zertifikat die Bestätigung erteilt, eine zertifizierte „Cochlea-Implantat-versorgende Einrichtung“ (CIVE) zu sein. Das Zertifikat wird durch die Zertifizierungsorganisation zugestellt, aber inhaltlich durch den Präsidenten der Deutschen Gesellschaft für Hals-Nasen-Ohren-Heilkunde, Kopf- und Hals-Chirurgie e. V. verantwortet und unterschrieben. Das Zertifikat wird sowohl für die CI-Versorgung von Kindern oder von Erwachsenen als auch für die CI-Versorgung von Kindern und Erwachsenen ausgestellt. Das schriftliche Zertifikat kann sowohl innerhalb der Klinik als auch in der Außenkommunikation verwendet werden. Darüber hinaus wird die Berechtigung zur Nutzung des Zertifizierungslogos für die Dauer der jeweiligen Zertifikatsgültigkeit (18 Monate bzw. 3 Jahre) vergeben. Das Logo kann von der erfolgreich zertifizierten Einrichtung ebenfalls für die interne und externe Kommunikation verwendet werden.

## Ergebnisse

Die Einrichtung der beschriebenen organisatorischen Strukturen und die Benennung der Mitglieder der Zertifizierungskommission erfolgten plangemäß durch das Präsidium der DGHNO-KHC ab November 2019. Die Zertifizierungskommission erarbeitete daraufhin bis Juli 2021 erfolgreich den Erhebungs- und Kennzahlenbogen. Dieser umfasst 35 Items zur Erfassung der Struktur‑, Prozess- und Ergebnisqualität auf Basis der aktuellen CI-Leitlinie und des CI-Weißbuchs (Tab. 1).

**Table 1:** 

Fragen-Nr.	Frage	Antwortmöglichkeiten	
1	Wird das Weißbuch als Grundlage für die Struktur und Arbeit Ihrer Cl-versorgenden Einrichtung (CIVE) verwendet?	Ja □	Nein □
10	Über wie viele Cl-spezialisierte Audiologen gemäß Qualifikationsprofil verfügt die CIVE?	Anzahl:	
27	Werden die geforderten Anteile am Versorgungsprozess durch Ihre CIVE erbracht? (# nicht delegierbar, * Delegation möglich)	– Präoperative Evaluation (#) – Operation (#) – Medizinische Kontrolle (#) – Audiologische Basis‑/Folgetherapie/Nachsorge (#) – Hörtherapeutische Basis‑/Folgetherapie/Nachsorge (*) – Sprachtherapeutische Basis‑/Folgetherapie/Nachsorge (*) – Technische Nachsorge (*)	
33	Wird ein CI-Jahresbericht veröffentlicht?	Ja □	Nein □
35	Ist die Teilnahme der CIVE am Register gewährleistet?	Ja □	Nein □

Der vollständige Erhebungs- und Kennzahlenbogen ist als Supplement verfügbar (siehe Zusatzmaterial Online)

Anfang 2021 erfolgte die Beauftragung der unabhängigen Zertifizierungsorganisation (Fa. ClarCert GmbH, Neu-Ulm, Deutschland) zur Umsetzung eines standardisierten Zertifizierungsprozesses unter wissenschaftlicher Leitung der DGHNO-KHC.

**Figure 4:**
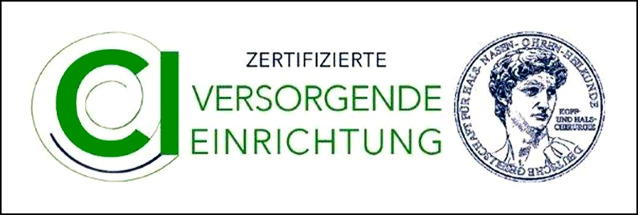

Nach Einrichtung eines Zertifikatserteilungsausschusses und Erstellung eines Zertifikat-Logos (Abb. 4) wurde das Zertifizierungsverfahren im August 2021 formal eröffnet und über die Homepage der DGHNO-KHC sowie über eine schriftliche Aussendung an die Mitglieder der DGHNO-KHC angekündigt. Ab September 2021 konnten Anträge zur Erteilung des Qualitätszertifikats CIVE gestellt werden.

### Zertifizierungsanträge und Zertifikatserteilung

Im Zeitraum von September bis Dezember 2021 wurden 29 Anträge zur Erteilung des CI-Zertifikats durch Kliniken gestellt. Im Zeitraum von Januar bis Dezember 2022 gingen 22 Anträge ein. Damit wurden in den ersten 16 Monaten seit Implementierung des Zertifizierungsverfahrens 51 Anträge auf Erteilung des Zertifikats gestellt.

Im Jahr 2021 wurden von den eingegangenen Anträgen 21 und im Jahr 2022 26 Anträge positiv beschieden. Damit lagen zum Ende des Jahres 2022, d. h. in den ersten 16 Monaten (September 2021 bis Dezember 2022) seit Implementierung des Zertifizierungsverfahrens, insgesamt 47 erteilte Zertifikate vor.

**Figure 5:**
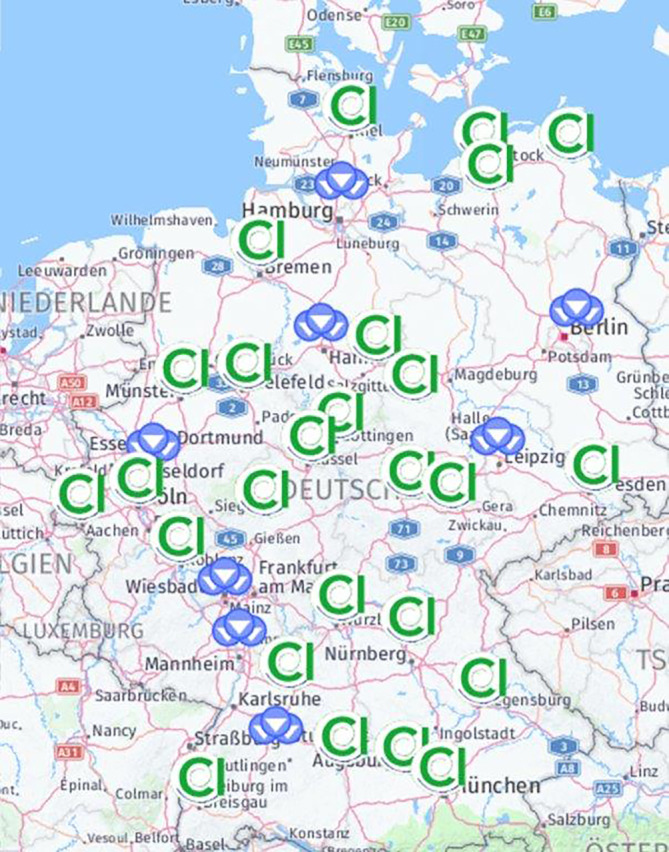

Im Jahr 2021 konnte bei einem Antrag und im Jahre 2022 bei 3 Anträgen aufgrund inkonsistenter Informationen oder Nichterfüllung der Vorgaben bislang kein Zertifikat erteilt werden. Eine Aufstellung der erfolgreich zertifizierten Einrichtungen wird kontinuierlich von der Zertifizierungsorganisation über deren Homepage erstellt [7]. Diese Aufstellung wird auf die Homepage der DGHNO-KHC verlinkt, um beides öffentlich zugänglich zu machen. Die Darstellung erfolgt in Form einer Landkarte mit örtlicher Markierung der CIVE in Deutschland und in Form einer Suchmaske (Abb. 5).

### Schulung der Fachexperten

Die Schulung der Fachexperten zur Vorbereitung der Vor-Ort-Auditierung erfolgte im März 2022. An dieser Schulung nahmen insgesamt 20 Personen mit der geforderten Grundqualifikation teil, die sich zuvor um die Teilnahme an dem Lehrgang beworben hatten. Von den 20 Teilnehmern wurden nach erfolgreicher Absolvierung des Lehrgangs 13 Personen für die Durchführung der Auditierung zu qualifizierten Fachexperten bzw. Auditoren benannt.

### Vor-Ort-Auditierung

Nachdem ab Oktober 2021 die ersten Zertifikate erfolgreich vergeben werden konnten, erfolgten wie geplant ab Juli 2022 die ersten Vor-Ort-Auditierungen durch die geschulten Fachexperten. Bis zum Ende des Jahres 2022 konnten bereits 13 Vor-Ort-Audits praktisch durchgeführt werden. Für das Jahr 2023 sind bislang weitere 27 Vor-Ort-Audits zur Aufrechterhaltung der Zertifikate terminiert. Die bisher erfolgten Vor-Ort-Audits zeigten eine hohe Übereinstimmung der zuvor anhand des Erhebungs- und Kennzahlenbogens gemachten Angaben der Kliniken, die durch die Fachexperten verifiziert wurden. In einzelnen Fällen wurden Empfehlungen für Verbesserungspotenziale ausgesprochen, die allerdings keine „kritischen Abweichungen“ darstellten und daher bisher einer Zertifizierung nicht im Wege standen (Tab. 2).

**Table 2:** 

	2021	2022	Gesamt
Anträge Erstzertifizierung (Offsite-Prüfung)	29	22	51
Erstellte Zertifikate (Offsite-Prüfung)	21	26	47
Nicht entscheidungsfähige oder nicht erteilte Zertifikate (Offsite-Prüfung)	1	3	4
Durchgeführte Vor-Ort-Prüfung für Erstzertifizierung	–	13	13

## Diskussion

In Deutschland hat sich über die letzten 30 Jahren ein stabiles System entwickelt, das im Hinblick auf die Entwicklung der Indikationsstellung zur CI-Versorgung als sehr innovativ betrachtet werden kann. Beispielhaft können hier die Themen bilaterale Versorgung [8], Versorgung einer einseitigen Taubheit [9, 10] sowie Versorgung bei Resthörigkeit durch elektrisch-akustische Stimulation (EAS) [11] genannt werden, die jeweils bedeutende Impulse aus Deutschland erhalten haben. Unabhängig von einem innovativen Umfeld existieren dennoch erhebliche Unterschiede in Bezug auf die Versorgungsprozesse, sodass bislang in Deutschland eine sehr heterogene und nicht-standardisierte Versorgungslandschaft besteht. Mit der Erstellung der CI-Leitlinie und des CI-Weißbuchs konnte unter Federführung der DGHNO-KHC erfolgreich ein einheitlicher wissenschaftlicher Konsens für die CI-Versorgung in Deutschland definiert werden.

Eine unabhängige Überprüfung der Umsetzung von Leitlinieninhalten war bislang, z. B. für Patienten, i. d. R. kaum möglich. So existieren auf der einen Seite in Deutschland klare Empfehlungen für die Struktur‑, Prozess- und Ergebnisqualität der CI-Versorgung, und auf der anderen Seite fehlte ein objektiver Nachweis der Einhaltung der Empfehlungen der Leitlinie. Diese Lücke sollte durch die Einführung des hier dargestellten Zertifizierungssystems von CI-versorgenden Einrichtungen (CIVE) geschlossen werden.

Die Zielsetzung des dargestellten Zertifizierungskonzepts zur CIVE besteht in der leitlinien- und weißbuchkonformen Umsetzung des CI-Versorgungsprozesses. Im Vergleich dazu beschreiben die zertifizierten „Audiologischen Zentren“ der DGA [12] Grundzüge einer integrierten Diagnostik und Versorgung komplexer Hörstörungen, ohne dabei ausschließlich den Einsatz von CI bzw. Hörimplantaten aufzuführen. Auch wenn sich in einzelnen Aspekten Parallelen in den Konzepten finden, weisen die beiden Ansätze doch erhebliche Unterschiede in ihrer Zielsetzung auf [13] und sind damit keinesfalls als äquivalent zu betrachten.

Die Erarbeitung der organisatorischen und strukturellen Grundlagen des Zertifizierungsprozesses begann bereits im Jahr 2017. Hier wurden wegweisende Grundsatzentscheidungen der DGHNO-KHC getroffen, um das Thema der Qualitätssicherung der CI-Versorgung auf der Grundlage medizinisch-wissenschaftlicher Erkenntnisse aktiv weiterzuentwickeln. Nachdem im Oktober 2020 die konsentierte 3. Fassung der Leitlinie zur CI-Versorgung in Deutschland durch die AWMF veröffentlicht wurde, konnte das Weißbuch „Cochlea-Implantat(CI)-Versorgung in Deutschland“ durch die DGHNO-KHC im Mai 2021 erstellt werden. Mit diesen Dokumenten lagen die wesentlichen Eckpfeiler zur Erarbeitung des Zertifizierungsprogramms vor.

Mit der im Rahmen dieser Arbeit dargestellten Konzeption und Struktur konnte das Zertifizierungsprogramm zwischenzeitlich sehr erfolgreich umgesetzt werden. Innerhalb von nur knapp 1,5 Jahren ist es gelungen, mit Stand Dezember 2022, 47 Kliniken für Hals-Nasen-Ohren-Heilkunde als CIVE zu zertifizieren. Durch das Zertifikat und die Verwendung des entsprechenden Logos wird eine einfache Erkennbarkeit der Einhaltung einer leitlinienkonformen Struktur‑, Prozess- und Ergebnisqualität in Bezug auf die CI-Versorgung für Patienten und Behandler erreicht. Außer Frage steht, dass diese Initiative erst am Anfang ihrer Umsetzung steht und weiterhin zahlreiche Herausforderungen existieren, die den Gegenstand zukünftiger Arbeit darstellen und im Folgenden erörtert werden sollen.

Die Anzahl von bisher 47 zertifizierten Kliniken kann aus unterschiedlichen Perspektiven bewertet und eingeordnet werden. Im Hinblick auf die relative Bewertung dieser Zahl sind verschiedene Faktoren zu berücksichtigen, wie z. B. die Einwohnerzahl Deutschlands. Diese liegt gegenwärtig bei etwa 84 Mio., sodass, rein rechnerisch, auf etwa 1,5 Mio. Bewohner eine zertifizierte Einrichtung kommt. Ob dieses Verhältnis angemessen ist oder einer Über- bzw. Unterversorgung entspricht, kann weder anhand der erhobenen Zahlen beurteilt noch bewertet werden. Dies gilt insbesondere auch in Bezug auf die internationale Versorgungssituation, für die es kaum verlässliche Referenzwerte gibt, an denen eine Orientierung derzeit sinnvoll möglich wäre.

Die hier dargestellte Arbeit stellt einen ersten Anhaltspunkt zur Anzahl der den Qualitätsstandards folgenden Einrichtungen in Deutschland dar. Berücksichtigenswert ist in diesem Zusammenhang, dass die Zielsetzung des Zertifizierungsprozesses zu keinem Zeitpunkt darin bestand, die Anzahl der Kliniken, die eine CI-Versorgung anbieten, zu limitieren oder indirekt eine Mindestmenge für die Versorgung einzuführen. Die Zielsetzung war vielmehr ausschließlich der objektive Nachweis der Erfüllung der Qualitätsstandards, der geltenden CI-Leitlinie und dem CI-Weißbuch entsprechend. Insofern ist eine Bewertung, ob es sich bei 47 Kliniken um eine hohe oder eine niedrige Zahl für die Versorgung der Bevölkerung in Deutschland handelt, nicht Gegenstand dieses Projekts.

Berücksichtigt man bereits existierende Erhebungen zur Versorgungsdichte in Deutschland, so ist eine frühere Arbeit, die dieses Thema aufgreift, zu nennen. In der 2020 von der DGHNO-KHC initiierten Untersuchung [14] wurden alle zu dem Zeitpunkt existierenden 170 HNO-Kliniken in Deutschland zur Durchführung einer CI-Versorgung befragt. In dieser Untersuchung gaben 70 Kliniken an, dass sie eine CI-Versorgung anbieten. Aus der Untersuchung geht aber auch hervor, dass die tatsächliche Anzahl der Kliniken, die eine CI-Versorgung anbieten, wahrscheinlich deutlich höher lag, da weniger als die Hälfte der Kliniken an der Umfrage teilgenommen haben. In dieser Arbeit wird daher eher von einer Anzahl von etwa 100 Kliniken ausgegangen. In Bezug auf diese Daten erscheint daher die Anzahl von bisher 47 zertifizierten Kliniken eher niedrig.

Berücksichtigt man allerdings den sehr kurzen Zeitraum (etwa 1,5 Jahre) des laufenden Zertifizierungsverfahrens, ist eine Anzahl von 47 Kliniken, die bereits erfolgreich zertifiziert wurden, wiederum eher als eine hohe Zahl zu betrachten. Diese Anzahl zeigt zum einen die große Akzeptanz des Zertifizierungssystems durch die Kliniken und zudem das hohe Interesse zur Teilnahme an diesem Verfahren. Über die Gründe, warum sich einzelne Kliniken bislang entschieden haben, nicht teilzunehmen, kann gegenwärtig nur spekuliert werden. Hier könnten unterschiedliche Faktoren, wie z. B. ein mangelndes Interesse am Zertifikat, die fehlende Unterstützung durch die Krankenhausverwaltung, eine organisatorische Verzögerung aufseiten der Antragsteller oder auch fehlende finanzielle oder personelle Ressourcen eine Nichtbeteiligung am Zertifizierungsverfahren bedingen. Möglicherweise werden aber auch gegenwärtig Anträge von einigen Einrichtungen nicht gestellt, da diese die geltenden Qualitätsaspekte der Leitlinie erkennbar nicht erfüllen würden. In diesem Szenario könnte sich eine Klinik bewusst entscheiden, momentan keinen Zertifizierungsantrag zu stellen, da dieser absehbar abgelehnt werden würde. Da alle eingegangenen Zertifizierungsanträge zwischenzeitlich entweder positiv entschieden, abgelehnt oder seitens der Antragsteller ergänzend bearbeitet werden müssen, ist das implementierte Zertifizierungssystem vollständig funktional und hatte weder einen positiven noch negativen Einfluss auf die Anzahl der inzwischen zertifizierten Kliniken.

Betrachtet man die hohen Anforderungen, die in Bezug auf Struktur‑, Prozess- und Ergebnisqualität durch die antragstellenden Einrichtungen erfüllt werden müssen, bei gleichzeitig großem Interesse der Kliniken, eine nach außen gerichtete, zertifizierte, qualitativ hochwertige CI-Versorgung belegen zu können, ergeben sich hieraus eine Reihe interessanter Aspekte. Im Rahmen der Vorbereitung einzelner Zertifizierungsanträge konnte, nicht zuletzt aufgrund der klar definierten Strukturvoraussetzungen einer CIVE, mit der jeweiligen Krankenhausadministration hinsichtlich notwendig zu ergänzender Ausstattungs- oder Personalmerkmale erfolgreich argumentiert werden. Diese aus den Reihen der Antragsteller rückgespiegelte Wahrnehmung kann als konstruktiver, positiver Effekt des Zertifizierungsprozesses betrachtet werden, da hier qualitätsrelevante strukturelle, apparative oder qualitative Lücken einer Klinik geschlossen werden konnten. Insofern ist die Einführung des Zertifizierungssystems für die CI-Versorgung nicht nur ein aktiver Nachweis bestehender Qualität einer Einrichtung, sondern hat bereits in diesem kurzen Zeitraum zu einer tatsächlichen Verbesserung der Versorgungsqualität in einigen Kliniken beigetragen.

Im Hinblick auf die Nutzung eines Zertifizierungssystems für weitere qualitätsrelevante Entwicklungen ist die kontinuierliche Erhebung klinisch relevanter Daten aus der Patientenbehandlung unabdingbar. Um zukünftig objektive Standards weiterzuentwickeln, kommt der Einrichtung von klinischen Registern eine besondere Bedeutung zu. Gerade im Nachgang der Problematik fehlerhafter Brustimplantate [15] existiert auch innerhalb der Gesundheitspolitik ein hohes Interesse an der Einführung von klinischen Registern. Durch das „Implantateregistergesetz – IRegG“ [16] wurden hierfür in Deutschland bereits die rechtlichen Grundlagen geschaffen. In diesem Gesetz ist, neben anderen Implantaten, auch das CI als ein Ziel zukünftiger Register benannt. Diese Perspektive berücksichtigend hat die DGHNO-KHC frühzeitig die Voraussetzungen zur Erstellung eines nationalen CI-Registers (Deutsches Cochlea-Implantat-Register: DCIR) erarbeitet, das sich gegenwärtig bereits in der praktischen Umsetzungsphase befindet (Registerbetreiber: INNOFORCE, Ruggell, Liechtenstein). Um zukünftig auch in dieser Beziehung handlungsfähig zu sein, ist eine der notwendigen Voraussetzungen zur Zertifikatserteilung an eine Klinik die dokumentierte Sicherstellung zur Teilnahme am nationalen CI-Register. Über diesen Schritt ist sichergestellt, dass eine Zertifizierung nur möglich ist, wenn eine Klinik auch klinische Daten zum CI-Register beiträgt. Eine ausschließliche Teilnahme am CI-Register ist hingegen auch ohne Zertifizierung möglich. Die Nichtteilnahme am CI-Register bedingt aber automatisch den Verlust des CIVE-Zertifikats. Hierdurch wird die zukünftige Weiterentwicklung der Qualitätsstandards sichergestellt, darüber hinaus werden wesentliche Aspekte der Ergebnisqualität, einschließlich möglicher Komplikationen oder Fehlentwicklungen der Versorgung, systematisch und deutschlandweit erhoben. Ebenfalls können über diesen Ansatz mögliche Evidenzlücken in der Erstellung von klinischen Leitlinien erkannt und wissenschaftlich basiert gefüllt werden.

Das hier präsentierte und in Deutschland erfolgreich eingeführte Zertifizierungssystem basiert auf den medizinisch-wissenschaftlichen Grundlagen der CI-Leitlinie. Es steht außer Frage, dass zukünftig weitere technische Entwicklungen (z. B. „Remote Fitting“) oder Veränderungen der Indikation, Veränderungen des Rehabilitationsprozesses (z. B. Selbstanpassung der Patienten) oder auch internetbasierte technisch-medizinische Verfahren (z. B. „Remote Check“) ihren Platz in der Versorgung von CI-Patienten finden werden. Es wird daher die zukünftige Aufgabe der DGHNO-KHC sein, unter Mitarbeit der beteiligten Fachgesellschaften, den jeweils geltenden medizinischen Standard zu erfassen und in eine zukünftige Version der CI-Leitlinie einzubringen. Insofern stellt das hier präsentierte Zertifizierungssystem ein universelles Instrument dar, um auch zukünftig struktur-, prozess- und ergebnisrelevante Aspekte in den Klinken zu erheben. Es steht ebenfalls außer Frage, dass es sich hierbei um ein dynamisches System handelt, das weitere Veränderungen und Anpassungen erfahren wird. Dennoch wird auch unter veränderten zukünftigen Qualitätsinhalten eine Zertifizierung zur transparenten Dokumentation unabdingbar sein und Relevanz haben.

Die Entwicklung von Leitlinien unterliegt auf internationaler Ebene keinen einheitlichen Regeln. Medizinrecht ist die Sache des jeweiligen Landes. Die Entwicklung von medizinischen Standards obliegt i. d. R. der jeweils führenden medizinischen Fachgesellschaft. Am Beispiel der CI-Versorgung ist dies i. d. R. die jeweilige HNO-Fachgesellschaft des betreffenden Landes. Die Voraussetzung zur Einrichtung eines Qualitätssicherungssystems mit kombinierter Zertifizierung ist die Erarbeitung einer Leitlinie. Über die weltweite Verbreitung und Nutzung von Leitlinien für die CI-Behandlung existieren bisher nur sehr wenige Daten. In einer kürzlich veröffentlichten Studie konnte für Europa in Bezug auf die Etablierung von Leitlinien und Registern ein sehr heterogenes Bild abgeleitet werden. Zum Zeitpunkt der Untersuchung existierten nur in 16 von 42 Ländern nationale Leitlinien für die CI-Versorgung [17]. Die Erstellung von nationalen Leitlinien ist aber eine zwingende Voraussetzung für die sinnvolle Etablierung von Qualitätssicherungsmaßnahmen mithilfe einer Zertifizierung. In einer realistischen Betrachtung muss allerdings festgestellt werden, dass sowohl die ökonomischen als auch die strukturellen Voraussetzungen zur Etablierung von Qualitätssicherungsmaßnahmen in verschiedenen Ländern erheblich voneinander abweichen, sodass der für Deutschland beschriebene Zertifizierungsansatz nicht zwingend ein für ein anderes Land umzusetzender Weg zur Qualitätssicherung sein muss. Dennoch kann das hier beschriebene methodische Vorgehen ein Beispiel dafür geben, einen landesindividuellen Ansatz zu erarbeiten.

Kritisch zu betrachten ist die in Deutschland gegenwärtig ausstehende Finanzierung des CI-Zertifizierungssystems. Die organisatorische Strukturierung, Erarbeitung der Standards und auch die praktische Umsetzung wurden ehrenamtlich durch die DGHNO-KHC erarbeitet. Die Kosten zur Teilnahme am Zertifizierungsverfahren (mehrere tausend Euro pro Jahr) werden derzeit ausschließlich durch die antragstellenden Einrichtungen getragen. Gegenwärtig unterscheidet die finanzielle Kompensation der CI-Leistungserbringung durch die Kostenträger nicht zwischen zertifizierten und nichtzertifizierten Einrichtungen. Eine klare Forderung an die Kostenträger muss daher sein, den nachweisbaren Aufwand zur Qualitätssicherung, einschließlich der Zertifizierung, im Interesse der Versicherten und Leistungserbringer zu kompensieren. Dies ist besonders kritisch vor dem Hintergrund der für 2023 erfolgten drastischen Reduktion der Vergütung einer CI-Operation (Diagnosis Related Groups, DRG: D01B). Die Wahrung der Qualität einer medizinischen Versorgung erfordert Prozesse und Strukturen, die mit einem objektiv messbaren (finanziellen) Aufwand einhergehen. Am Beispiel der CI-Versorgung zeigt der dargestellte Zertifizierungsprozess, dass nicht nur die Umsetzung und Einhaltung medizinischer Qualitätsstandards, sondern auch die unabhängige Überprüfung derselben einen erheblichen Aufwand bedingt und damit Zeit und v. a. auch Geld erfordert. Hierfür müssen die notwendigen finanziellen Mittel für die Kliniken durch die Kostenträger zwingend bereitgestellt werden. Dies gilt sowohl für den CI-Zertifizierungsprozess als auch für das CI-Register.

## Ausblick

Die Versorgung von hochgradig schwerhörigen oder ertaubten Menschen mit einem CI ist eine sehr erfolgreiche Therapie, die zugleich einen komplexen und lebenslangen Versorgungsprozess erfordert. Ein standardisierter und strukturierter Versorgungsprozess stellt daher die unabdingbare Voraussetzung für eine optimale Hörrehabilitation mit CI dar. Im Oktober 2020 wurde die aktuelle AWMF-Leitlinie zur CI-Versorgung verabschiedet, die zentrale Aspekte der Qualitätssicherung, wie Strukturqualität, Prozessqualität und Ergebnisqualität umfasst. Diese Leitlinie beschreibt damit den derzeit einheitlich in Deutschland geltenden medizinischen Standard in der CI-Versorgung. Auf Initiative des Präsidiums der DGHNO-KHC wurde auf Basis dieser Leitlinie ein Weißbuch erstellt und ein Zertifizierungssystem eingeführt, um die Umsetzung dieser Leitlinie standardisiert einrichtungsbezogen zu überprüfen und öffentlich zugänglich zu machen. Unter Einbindung einer unabhängigen Zertifizierungsorganisation kann nun den beantragenden Kliniken durch die Erteilung des Qualitätszertifikats CIVE die erfolgreiche Umsetzung der Leitlinien- und Weißbuchinhalte bescheinigt werden.

Die seit der Implementierung über etwa 1,5 Jahre gewonnenen Erfahrungen belegen eindrucksvoll den Beitrag dieses methodischen Ansatzes zur transparenten Qualitätssicherung in der Medizin. Die hohe Akzeptanz, die starke Unterstützung durch die Kliniken sowie die gute Funktionalität des dargestellten Systems zeigt das enorme Potenzial zur Qualitätssicherung in der CI-Versorgung. Ein auf einer wissenschaftlichen Leitlinie basierendes und durch die fachlich führende medizinisch-wissenschaftliche Fachgesellschaft (DGHNO-KHC) umgesetztes Zertifizierungssystem könnte auch für weitere Bereiche der Medizin oder andere Länder beispielgebend zur transparenten Umsetzung von Qualitätsstandards sein.

## Fazit für die Praxis

- Zusammenfassend demonstriert die hier präsentierte Arbeit, dass innerhalb von etwa 1,5 Jahren die Konzeptionierung, Strukturierung und praktische Umsetzung eines Zertifizierungssystems zur Qualitätssicherung der Versorgung mit einem Cochlea-Implantat (CI) in Deutschland sehr erfolgreich durch die Deutsche Gesellschaft für Hals-Nasen-Ohren-Heilkunde, Kopf- und Hals-Chirurgie e. V. (DGHNO-KHC) umgesetzt werden konnte.
- Es wurde das Qualitätszertifikat „Cochlea-Implantat-versorgende Einrichtung“ (CIVE) entwickelt.
- Die hohe Akzeptanz des CIVE-Zertifizierungssystems belegt die Unterstützung und Funktionalität des dargestellten Systems, sodass die entwickelte Struktur möglicherweise auch beispielgebend für andere Länder oder auch andere Bereiche der Medizin sein kann.

## Supplementary Information





## References

[1] Dazert S, Thomas JP, Loth A, Zahnert T, Stöver T (2020). Cochlear Implantation. Dtsch Arztebl Int.

[2] Zeh R, Baumann U (2015). Stationäre Rehabilitationsmaßnahmen bei erwachsenen CI-Träger. Ergebnisse in Abhängigkeit von der Dauer der Taubheit, Nutzungsdauer und Alter. HNO.

[3] Issing C, Baumann U, Pantel J, Stöver T (2020). Cochlear implant therapy improves the quality of life in older patients—A prospective evaluation study. Otol Neurotol.

[4] https://register.awmf.org/de/leitlinien/detail/017-071. Zugegriffen: 01. Jan. 2023

[5] https://www.ci-register.de/wp-content/uploads/DGHNO_Weissbuch_CI-Versorgung.pdf. Zugegriffen: 01. Jan. 2023

[6] https://www.gesetze-im-internet.de/mpbetreibv/BJNR176200998.html. Zugegriffen: 01. Jan. 2023

[7] https://www.clarmap.com (Zertifizierungssystem: CIVE) oder „https://ddei5-0-ctp.trendmicro.com:443/wis/clicktime/v1/query?url=www.clarmap.com&umid=D04808CD-F25D-C805-831F-841D0E2FC1DC&auth=92748e9654136e9487f01ed43828d21d409b58ea-4848f95a2ffa15de76ba11c8c132ac12d66a008e“. Zugegriffen: 01. Jan. 2023

[8] Müller J, Schön F, Helms J (2002). Speech understanding in quiet and noise in bilateral users of the MED-EL COMBI 40/40+ cochlear implant system. Ear Hear.

[9] Jacob R, Stelzig Y, Nopp P, Schleich P (2011). Audiologische Ergebnisse mit Cochlear implant bei einseitiger Taubheit. HNO.

[10] Arndt S, Aschendorff A, Laszig R, Beck R, Schild C, Kroeger S, Ihorst G, Wesarg T (2011). Comparison of pseudobinaural hearing to real binaural hearing rehabilitation after cochlear implantation in patients with unilateral deafness and tinnitus. Otol Neurotol.

[11] von Ilberg C, Kiefer J, Tillein J, Pfenningdorff T, Hartmann R, Stürzebecher E, Klinke R (1999). Electric-acoustic stimulation of the auditory system. New technology for severe hearing loss. ORL J. Otorhinolaryngol. Relat. Spec..

[12] https://www.dga-ev.com/fileadmin/daten/downloads/Audiologische_Zentren_Konzept_2016_final.pdf. Zugegriffen: 1. Jan. 2023

[13] Baumann U (2022). CI-versorgende Einrichtung (DGHNO) und Audiologisches Zentrum (DGA). Z Audiol.

[14] Stöver T, Zeh R, Gängler B (2020). Regionale Verteilung der Cochlea-Implantat (CI)-versorgenden Einrichtungen in Deutschland. Laryngorhinootologie.

[15] Lampert FM, Schwarz M, Grabin S, Stark GB (2012). The “PIP scandal”—complications in breast implants of inferior quality: state of knowledge, official recommendations and case report. Geburtshilfe Frauenheilkd.

[16] http://www.gesetze-im-internet.de/iregg/BJNR249410019.html. Zugegriffen: 1. Febr. 2023

[17] Loth A, Vazzana C, Leinung M, Guderian D, Issing C, Baumann U, Stöver T (2022). Quality control in cochlear implant therapy: clinical practice guidelines and registries in European countries. Eur Arch Otorhinolaryngol.

