# Supplementary material for: Conception and implementation of a certification system for quality control of cochlear implant treatment in Germany. German version
Source: HNO. 2023 Apr 28;71(6):396–407. [Article in German] doi: 10.1007/s00106-023-01305-x (PMC10234877; doi:10.1007/s00106-023-01305-x)
Supplement: Supplementary file 1 [file 106_2023_1305_MOESM1_ESM.pdf]

Deutsche Gesellschaft für
Hals-Nasen-Ohren-Heilkunde,
Kopf- und Hals-Chirurgie e.V.

Erhebungs- und Kennzahlenbogen

zur Erstzertifizierung von Cochlea-Implantat(CI)-versorgenden Einrichtungen

**Inkraftsetzung am 07.07.2021
durch die Zertifizierungskommission**

© 2021
Stand: N1 (25.08.2022)

Präambel

Die Anforderungen der Zertifizierung von CI-versorgenden Einrichtungen (CIVE) beruhen auf dem Weißbuch der Cochlea-Implantat(CI)-Versorgung und werden mit diesem Erhebungs- und Kennzahlenbogen erhoben.

Abkürzungsverzeichnis

CI	Cochlea-Implantat
CIVE	CI-versorgende Einrichtung (implantierende HNO-Klinik)
DGHNO-KHC	Deutsche Gesellschaft für Hals-Nasen-Ohren-Heilkunde, Kopf- und Hals-Chirurgie
HNO	Hals-Nasen-Ohren-Heilkunde
MTA-F	Medizinisch-technische Assistent/in für Funktionsdiagnostik
DGA	Deutsche Gesellschaft für Audiologie
MPSV	Medizinprodukte-Sicherheitsplanverordnung
MPBetreibV	Medizinprodukte-Betreiberverordnung
MPG	Medizinproduktegesetz

Ausfüllhinweise

Sofern eine Anforderung nicht erfüllt wird, ist eine Kommentierung durch die Einrichtung notwendig.

Eingaben durch das Zentrum erforderlich, tlw. mit Dropdown-Menü

Eingabe durch Einrichtung optional

Anforderung scheint nicht erfüllt, bitte begründen

Keine Eingabe erforderlich

Erklärung

Zur Vereinfachung und leichteren Lesbarkeit wird im Folgenden für die einzelnen Personenkategorien nur die männliche Form verwendet.
Bitte verwenden Sie, sofern vorhanden, die Dropdown-Funktion. Diese Datei wurde auf einem Windows-Rechner mit Microsoft Office 2013 erstellt.
Die Verwendung von älteren Office-Versionen oder anderen Kalkulationsprogrammen kann unter Umständen zu Einschränkungen und Funktionalitätsverlusten fü

Erhebungs- und Kennzahlenbogen zur Erstzertifizierung als Cochlea-Implantat(CI)-versorgende Einrichtung gemäß den Anforderungen der DGHNO-KHC

Legende

- Eingabe durch Einrichtung optional
- Eingabe durch Einrichtung erforderlich

Stammdaten

Reg. Nr.	CIVE-
Einrichtungsname	
Straße	
Hausnummer	
PLZ	
Ort	
Ärztliche Leitung	
Einrichtung mit Kinderversorgung	

Weitergehende Beschreibungen zur Darlegung der Anforderungserfüllung

1. Allgemeine Aspekte der CI-Versorgung

Nr.	Kapitel Weißbuch	Anforderung	Anforderungserfüllung	Kommentierung durch Einrichtung
1	---	Wird das Weißbuch als Grundlage für die Struktur und Arbeit Ihrer CI-versorgenden Einrichtung (CIVE) verwendet?		
2	1.	Wird die Verantwortung für den Gesamtprozess der CI-Versorgung durch die CIVE getragen?		
3	1.	Werden alle Teilschritte des Versorgungsprozesses (Präoperative Evaluation, Operation, Basistherapie (Erstanpassungsphase), Folgetherapie (Rehabilitation), Nachsorge) von der CIVE verantwortlich angeboten?		
4	--	Erfolgt an der CIVE die CI-Versorgung von Erwachsenen?		
5	--	Erfolgt an der CIVE die CI-Versorgung von Kindern?		

2. Strukturelle Voraussetzungen

Nr.	Kapitel Weißbuch	Anforderung	Anforderungserfüllung	Kommentierung durch Einrichtung
6	2.1	Wird ein interdisziplinäres Expertenteam / Kooperationsstruktur vorgehalten (gemäß Weißbuch Kap. 2.1)?		
7	3.1.6	Findet eine strukturierte und dokumentierte interdisziplinäre Fallkonferenz statt?		
8	3.2.2	Stehen durchgehend Konsiliardienste für Intensivmedizin, Neurochirurgie, Neuroradiologie und Pädiatrische Anästhesiologie und Intensivmedizin zur Verfügung?		

3. Personelle Mindestausstattung

Nr.	Kapitel Weißbuch	Kennzahl	Soll	Ist	Anforderungserfüllung	Kommentierung durch Einrichtung
9	2.3	Über wie viele HNO-Fachärzte, die auf CI spezialisiert sind, verfügt die CIVE?	2		Eingabe erforderlich	
10	2.3	Über wie viele CI-spezialisierte Audiologen gemäß Qualifikationsprofil, verfügt die CIVE?	1		Eingabe erforderlich	
11	2.3	Über wie viele Hörtechniker gemäß Qualifikationsprofil verfügt die CIVE?	1		Eingabe erforderlich	
12	2.3	Über wie viele MTA-F oder Audiologie-Assistent/-innen verfügt die CIVE?	2		Eingabe erforderlich	
13	2.3	**Bei Kinderversorgung:** Über wie viele Fachärzte für Phoniatrie & Pädaudiologie verfügt die CIVE?	1		Eingabe erforderlich	
14	2.3	Über wie viele Therapeuten der sprach- / sprechtherapeutischen Berufsgruppen verfügt die CIVE?	2		Eingabe erforderlich	

4. Apparative Voraussetzung

Nr.	Kapitel Weißbuch	Anforderung	Anforderungserfüllung	Kommentierung durch Einrichtung
15	2.5	Verfügt die CIVE über Hard- und Software zur Überprüfung konventioneller Hörgeräte?		
16	2.5	Verfügt die CIVE über Hard- und Software zur Anpassung für mind. 3 verschiedene CI-Implantatsysteme?		
17	2.5	Verfügt die CIVE über eine Messanlage für klick- und frequenzspezifische BERA, ASSR und CERA (DIN EN 60645-7:2010-08) (mit der Möglichkeit zur Messung in Narkose/Sedierung bei Kinderversorgung)?		
18	2.5	Verfügt die CIVE über die Möglichkeit zur Elektrocochleographie (mit der Möglichkeit zur Messung in Narkose/Sedierung bei Kinderversorgung)?		
19	3.2.2	Steht eine Röntgenuntersuchung zur intraoperativen Lagekontrolle des CI zur Verfügung?		
20	3.2.3	Steht ein Hirnnervenmonitoring / Facialismonitoring intraoperativ zur Verfügung?		
21	3.2.3	Steht eine E-Bera intraoperativ zur Verfügung?		

5. Mindestpatientenzahlen

Nr.	Kapitel Weißbuch	Anforderung	Anforderungserfüllung	Kommentierung durch Einrichtung
22	2.6	Werden an der CIVE mind. 1000 Routine-Audiometrie-Untersuchungen pro Jahr durchgeführt?		
23	2.6	Werden an der CIVE mind. 100 spezielle audiologischen Untersuchungen pro Jahr durchgeführt? (z. B. Abklärung CI-Indikation und pädaudiologische Diagnostik bei Kinderversorgung)		

6. Prozessablauf				
**Nr.**	**Kapitel Weißbuch**	**Anforderung**	**Anforderungserfüllung**	**Kommentierung durch Einrichtung**
24	3.1.1	Ist die Durchführung der Hörgeräteoptimierung in der CI-Versorgung gewährleistet?		
25	3.2.2	Werden Implantate von mind. 3 verschiedenen Herstellern angeboten?		
26	3.2.4	Wird die CI-Versorgung unter vollstationären Bedingungen durchgeführt?		
27	3.3	Werden die geforderten Anteile am Versorgungsprozess durch Ihre CIVE erbracht?	Bitte geben Sie in der rechten Spalte mittels Klick auf 'ja' bzw. 'nein' an, ob die entsprechende Versorgung durch Ihre CIVE erbracht wird.	Präoperative Evaluation: ja / nein<br>Operation: ja / nein<br>Basistherapie: Audiologische Basistherapie: ja / nein; Hörtherapeutische Basistherapie: ja / nein; Sprachtherapeutische Basistherapie: ja / nein; Medizinische Kontrolle: ja / nein<br>Folgetherapie: Audiologische Folgetherapie: ja / nein; Hörtherapeutische Folgetherapie: ja / nein; Sprachtherapeutische Folgetherapie: ja / nein; Medizinische Kontrolle: ja / nein<br>Nachsorge: Audiologische Nachsorge: ja / nein; Hörtherapeutische Nachsorge: ja / nein; Sprachtherapeutische Nachsorge: ja / nein; Medizinische Kontrolle: ja / nein; Technische Nachsorge: ja / nein<br>Grau hinterlegt: in der CIVE zu erbringende Prozessanteile (nicht delegierbar)<br>Weiß hinterlegt: unter Verantwortung der CIVE Delegation von Prozessanteilen optional möglich
28	3.3	Wird für erwachsene Patienten die hörtherapeutische (und ggf. die sprachtherapeutische) Basistherapie, Folgetherapie (Rehabilitation) und Nachsorge verantwortlich eingeleitet?		
29	3.3.1	Wird für Kinder eine interdisziplinäre, pädagogisch und hör-sprachtherapeutisch orientierte Rehabilitation (Folgetherapie) verantwortlich eingeleitet?		
30	3.5	Wird die jährliche Nachsorge durch die CIVE angeboten?		

7. Qualitätssicherung				
**Nr.**	**Kapitel Weißbuch**	**Anforderung**	**Anforderungserfüllung**	**Kommentierung durch Einrichtung**
31	2.7	Erfolgt die Wahrnehmung der Aufgaben unter Beachtung der relevanten Verordnungen (MPBetreibV, MPSV, MPG, Schulungen und Re-Zertifizierungen des Personals)?		
32	2.7	Wird ein CI-Jahresbericht erstellt?		
33	4.1	Wird der CI-Jahresbericht / Qualitätsbericht veröffentlicht?		
34	4.1	Existiert bereits eine CI-Datenbank in der CIVE?		
35	4.2	Ist die Teilnahme der CIVE am Register gewährleistet?		
